# Implant Treatment in Patients With Autoimmune Diseases: A Systematic Review and Analysis of Studies

**DOI:** 10.7759/cureus.67617

**Published:** 2024-08-23

**Authors:** Katia Sarafidou, Maria Lekatsa, Amalia Michou, Athina Bakopoulou, Athanasios Poulopoulos, Dimitrios Andreadis

**Affiliations:** 1 Prosthodontics, School of Dentistry, Faculty of Health Sciences, Aristotle University of Thessaloniki, Thessaloniki, GRC; 2 Dental Surgery, School of Dentistry, Faculty of Health Sciences, Aristotle University of Thessaloniki, Thessaloniki, GRC; 3 Oral Medicine/Pathology, School of Dentistry, Faculty of Health Sciences, Aristotle University of Thessaloniki, Thessaloniki, GRC

**Keywords:** prosthodontics, survival rate, dental implants, lupus erythematosus, epidermolysis bullosa, sjogren’s syndrome, lichen planus, autoimmune diseases

## Abstract

Dental implants provide a reliable solution for edentulous patients with autoimmune diseases improving quality of life. The present systematic review aimed to determine whether autoimmune diseases with oral manifestations could affect the survival rate of dental implants. A systematic search was conducted following the Preferred Reporting Items for Systematic Reviews and Meta-Analyses guidelines (PRISMA), using Google Scholar and PubMed electronic databases, between the year 2000 and February 2024. The eligibility criteria included human studies, in English language reporting on patients with autoimmune diseases treated with dental implants. Nineteen studies were included: nine on oral lichen planus (OLP), four on Sjögren's syndrome (SS), five on epidermolysis bullosa (EB), and one on lupus erythematosus (LE). A total of 389 implants in 142 patients with OLP showed a survival rate (SR) of 94.6%, while 316 implants in 111 patients with SS had a survival rate of 95.8%. In 31 patients with EB, 181 implants were placed with a survival rate of 99.5%, and 12 implants were placed in five patients with LE with a survival rate of 100% after one year of function. Despite the heterogeneity and methodological limitations of most of the studies, the results showed that dental implant survival rates were comparable to those reported in the general population. This review suggested that dental implants are a viable treatment option for patients with autoimmune diseases. Nevertheless, proper daily oral hygiene and long-term follow-up are decisive factors for the long-term maintenance of dental implants.

## Introduction and background

Nowadays, dental implants are widely used to successfully replace missing teeth. Implant treatment improves masticatory function and quality of life for edentulous or partially edentulous patients and is related to a success rate of almost 90-95% after 10 years of function [1]. At the same time, the immune system of the host is a determinant for the process of osseointegration by balancing the inflammatory response as part of the foreign body reaction to dental implants [2]. Osseointegration refers to a structural and functional connection between bone and dental implant without the existence of fibrous connective tissue [3]. Through osseointegration, implants become part of the host’s body and may carry occlusal loads to restore the patient’s chewing ability after loss of teeth. Nevertheless, specific medical conditions or chronic illnesses may interfere with the process of osseointegration, jeopardizing thus the intraoral survival of implants. There is no sufficient data in the literature with regard to which conditions negatively impact osseointegration and to which extent this impact may appear. In patients with autoimmune dermato-mucosal diseases, the oral epithelium and the connective tissue show significant changes [4]. Such changes may result in disconnection between the human tissue and the implant surface, accompanied by an increasing risk of contamination, leading to implant failure [5]. At the same time, the pharmacological treatment of chronic diseases further complicates the survival of dental implants since bone healing in the human body, or other inflammatory processes, may be altered through medication [6].

There is an increasing percentage of patients facing autoimmune diseases nowadays [7]. Conditions such as Sjogren’s syndrome (SS), oral lichen planus (OLP), epidermolysis bullosa (EB), or lupus erythematosus (LE) present manifestations in the oral cavity, which incommode the masticatory function and speaking ability of patients. Such patients may also suffer from tooth loss, which raises the question of which type of therapy - conventional prosthodontics or implant treatment - is preferable in such situations. The modified inflammatory response in such diseases, as well as the side effects of the prescribed medication, reasonably lead dental practitioners to be reluctant to provide complex prosthetic treatment plans, such as those involving dental implants, in such cases. On the other hand, such patients may benefit from implant-supported prostheses since the latter provide optimal comfort in the rehabilitation of the edentulous oral areas. Studies published on this issue are rare to find in literature and do not address the matter of modern medication prescribed or the newest treatment plans adopted for those diseases [8,9].

The present systematic review aimed to determine whether autoimmune diseases with oral manifestations, such as OLP, SS, EB, and LE, may affect the survival rate of dental implants.

## Review

Materials and methods

The conduct of this systematic review was based on the guidelines set by PRISMA (Preferred Reporting Items for Systematic Reviews and Meta-analyses) [10]. Data were collected on patients with oral mucosal diseases rehabilitated with dental implants. The focused question was elaborated by using the PICO format as follows: P for Population (patients receiving dental implants), I for Interest Variable (patients with autoimmune disease with oral manifestations), C for Comparison (healthy subjects), O for Outcome (biological complications and implant survival). 

Search Strategy and Study Selection

The search was conducted using two different electronic databases: Google Scholar and PubMed of the US National Library of Medicine, between the year 2000 and February 2024. The MeSH terms used were ((oral lichen planus) AND (dental OR oral implants OR dental implant rehabilitation)),((Sjogren’s syndrome) AND (dental OR oral implants OR dental implant rehabilitation)),((lupus erythematosus AND (dental OR oral implants OR dental implant rehabilitation)), ((epidermolysis bullosa) AND (dental OR oral implants OR dental implant rehabilitation)), ((pemphigus) AND (dental OR oral implants OR dental implant rehabilitation)), ((pemphigoid) AND (dental OR oral implants OR dental implant rehabilitation)). The articles retrieved were in English language only. Eligibility criteria included clinical human studies, either randomized or not, reporting on patients with oral mucosal diseases, rehabilitated with implant-retained and/or implant-supported oral prostheses, and a minimum follow-up of one year. Exclusion criteria were case reports, animal studies, in vitro studies, and review papers. Two independent reviewers performed the search. A hand search was also conducted in the reference lists of the retrieved articles, in order not to miss any relevant publications. The second step of the search included the retrieval of the abstracts of the initially selected articles, by each of the two primary investigators, for potential selection in this systematic review following the inclusion criteria initially set. The rest of the authors were called in to assist when there was any disagreement between the principal investigators. Throughout the search period, all participating authors were informed about the selected articles. Downloading of the abstracts of the approved papers was then performed and independently assessed by each of the principal investigators. The full text of the article was retrieved if the abstract satisfied the initially set criteria. In cases where the abstract did not provide sufficient information, the full text was also retrieved. After a meticulous reading of all retrieved full texts, the final articles were selected, based on the fulfillment of the inclusion criteria. 

Data Extraction

The authors independently extracted data using specially designed data extraction forms. Any disagreements were resolved by discussion. For each of the identified studies included, the following data were then extracted: the patient’s age, oral mucosal disease, number of implants, implant type, survival rate, complications, type of implant-supported prosthesis, and follow-up period. The authors of the included studies were contacted in cases where data were missing.

Results

**Figure 1:**
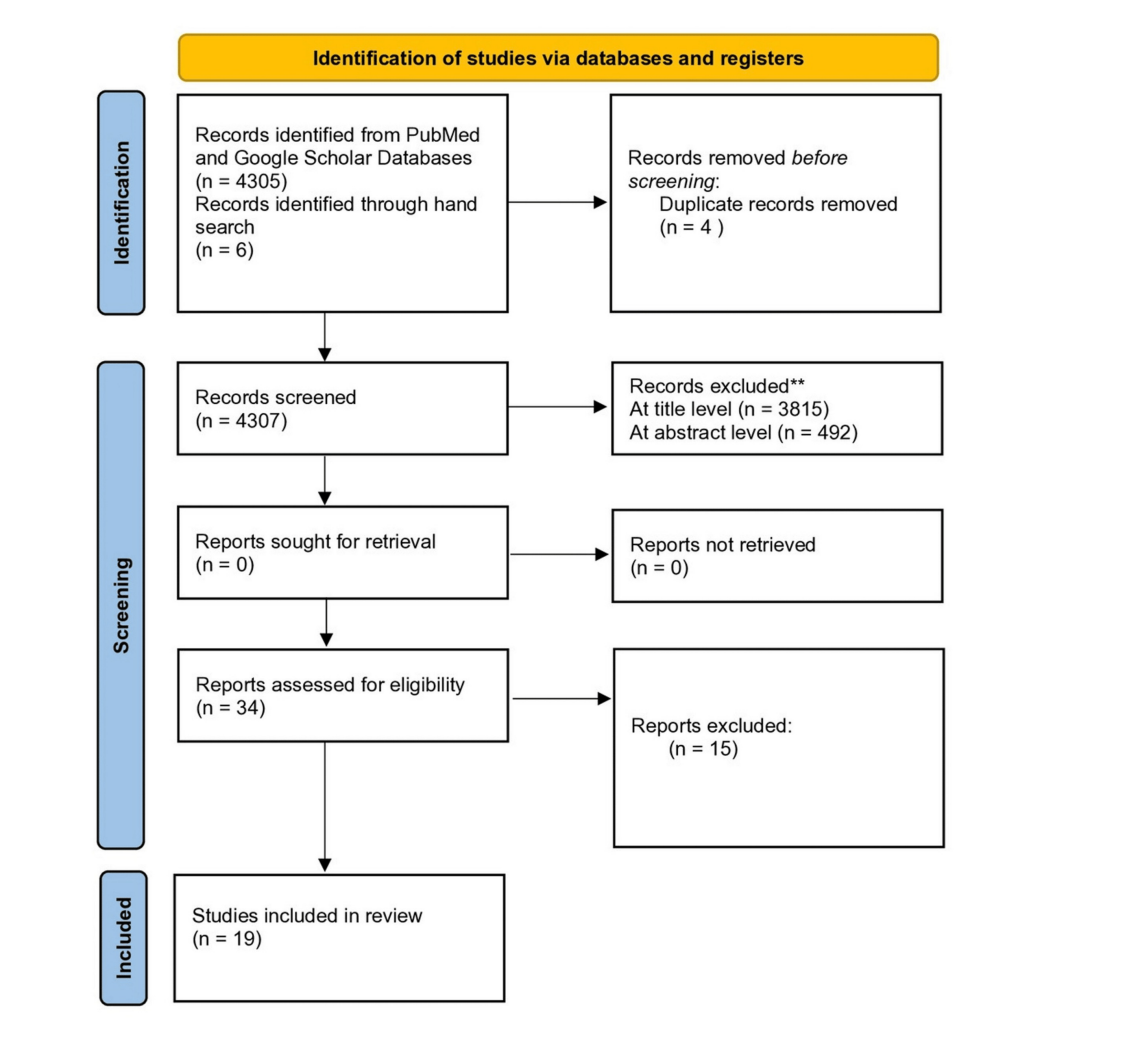
PRISMA 2020 flow diagram for new systematic reviews that included searches of databases and registers only

A total of 34 articles were selected for full-text analysis from an initial yield of 4,307 studies. The PRISMA flowchart is given in Figure 1. After discussion and further evaluation between the authors, another 15 full-text articles were excluded and not analyzed further. Nineteen studies met the inclusion criteria and were included in the systematic review.

Data were analyzed and synthesized in a table, including the following parameters: lead author, year of publication, type of study, reported disease, number of participants, gender, age, number of implants placed, implant type, survival rate, reported complications, type of implant-prosthesis, and follow-up period (Table 1). The studies referred to patients suffering from OLP, LE, EB, and SS. Of the 19 clinical included studies, four followed a prospective investigation protocol, eight a retrospective one, and seven case series were also included. The total number of implants placed in all patients was 898. The overall survival rate was 96.2% for the follow-up of the first year.

**Table 1:** Selected studies included in the review and clinical data Abbreviations: RS: retrospective study, CSS: cross-sectional study PS: prospective study, CS: case series, OLP: oral lichen planus, SS: Sjogren's syndrome, LE: lupus erythematosus, EB: epidermolysis bullosa, DEB: dystrophic epidermolysis bullosa, RDEB: recessive dystrophic epidermolysis bullosa, PIM: peri-implant mucositis, DG: desquamative gingivitis, PI: peri-implantitis, IM: implant mobility, IL: implant loss, PIBL: peri-implant bone loss, NR: not reported, PI: plaque index, BI: bleeding index, GI: gingival index, PD: probing depth, MBL: marginal bone loss, IR: inflammation rate, FSR: fixed screw retained, FC: fixed cemented, RIS: removable implant supported

	Author	Publication year	Type of study	Disease	Number of patients	Age (mean or range)	Number of implants	Implant type	Survival rate	Complication	Prosthetic rehabilitation	Follow-up
1.	Czerninski [11]	2011	RS	OLP	14	59.5	54	-	100%	-	-	12-24 months
2.	Hernandez [12]	2012	PCS	OLP	18	53.7	56	Ti Unite(Nobel Biocare S.A Gothenburg,Sweden 4.0,5.0,Nobel Direct 3mm	100%	44.6% PIM, 10.7% PI	FSRP except Nobel Direct patients-FCS	53.5 months
3.	Lopez-Jornet [13]	2014	CSS	OLP	16	64.5(44-76)	56	-	96.4%	PI 25% PIM 17.86%	3 RIS 13 FC	12-120 months
4.	Anitua [14]	2018	RS	OLP	23	58	66	Short implants (<8.5mm)	98.5%	PIBL 0.96 mesially,0.99 distally, 1 IL	66 RIS 39 FSR 27 FC	24-124 months
5.	Khamis [15]	2018	PS	OLP	42	-	-	-	100%	-	-	4 years
6.	Aboushelib [16]	2016	PS	OLP	23	46-68	97	TSV Zimmer titanium Rental	73.6%	IM,IL (42 implants)	-	3 years
7.	Esposito [17]	2003	CS	OLP	2	72.78	4	4.1x8mm solid-screw, Straumann	100%	-	RIS	18 months
8.	Oczakir [18]	2005	CS	OLP,SS	1 LP 1 SS	74 LP 63.5 SS	4 LP 4 SS	ITI implants	100%	NR	FC	LP-6 years SS-2 years
9.	Reichart [19]	2006	CS	OLP	3	70	10	-	100%	-	-	3 years
10.	Maarse [20]	2022	PCS	SS	17	18-80	37	-	100%	-	FSR	18 months
11.	Korfage [21]	2015	CS	SS	50	67+/-8	140	-	97%	IL (%) 4 (3%) PI indices PI (0-3) 1.0 Calculus(0-1) 0.0 BI(0-3) 1.5 GI(0-3) 0.5 PD(mm) 3.5 MBL 0.89 PIM(patients,%) 47 (94%) (implants,%) 102(73%) PI(patients,%) 7 (14%)(implants,%) 16(11%)	27 SC 36 overdentures 2 FPP 1 FAFP	46 months/3.8 years
12.	Albrecht K [22]	2016	CS	SS	32	64.5	104	-	95.2%	4 removed implants 1 replaced implant	RIS	4.9+/-5.4 years
13.	Siddiqui [23]	2017	RS	SS	11	63-75	23	-	87%	IF:3, IM:2, erythema of surrounding implant area, vertical BL, and horizontal ridge deficiency after implant placement.	RIS	4.9+/-5.4 years 40 months
14.	Peñarrocha-Diago M/Serrano [24]	2000	CS	EB	4	26-35	15	-	100%	-	-	1-4 years
15.	Peñarrocha-Oltra D/Penarrocha - Diago [25]	2011	RS	EB	6	24-27	32	-	100%	-	8 FSR	22.9 (range 12-48) months after prosthetic loading
16.	Peñarrocha-Oltra D/Aloy-Prosper [26]	2012	RS	EB	4	27-44 years	23	23 implants, of which 18 (10 Defcon and 8 ITI) had peri-implant defects (dehiscence or fenestrations) with 2 to 8 (average 3.5)	100%	-	FC	12 months(12-48)
17.	Agustín-Panadero R/Serra-Pastor [27]	2019	CS	EB	4	20-52 years	31	Angled screws (Axis; Phibo Dental Solutions) were used for this purpose and were inserted at an angle of 25 degrees and tightened to 20 to 25 Ncm	100%	BI: 74.2%, IR: 58.0% displacement in 22.6% of the implants	FSR	4 years
18.	Peñarrocha-Oltra D/Agustin Panadero [28]	2020	RS	EB	13	20-59	80	-	97.5%	IL:2 IR of PI tissues:50% PI on the PI prosthesis:85% In turn, PI PD was maintained at 1-3 mm in 96.2% of the implants, though 52.5% of the implants showed 0 mm retraction of the PI mucosa PI BL after 7.7 years of follow-up was 1.65 0.54 mm	FSR FC RIS	2-15 years
19.	Mozzati [29]	2021	RS	LE	5	57	12	Calcium ions-modified surface	BL 0.49 dehiscence		-	5 years

Five studies reported on patients suffering from SS treated with a total number of 316 implants. The survival rate for implants placed in patients with SS was 95.8% for a follow-up of 18 months. OLP was found in nine of the included studies, while the total number of implants placed in those patients was 389 and showed a survival rate of 94.6%. Only one study on patients with LE met the inclusion criteria and was included in the review. In this study, 12 implants were placed with a survival rate of 100% after five years. Finally, the literature search revealed five publications reporting on patients with EB receiving dental implants. A total of 181 implants were inserted in those patients, and the total survival rate was 99.5%. 

Assessment of the quality of the included studies

**Figure 2:**
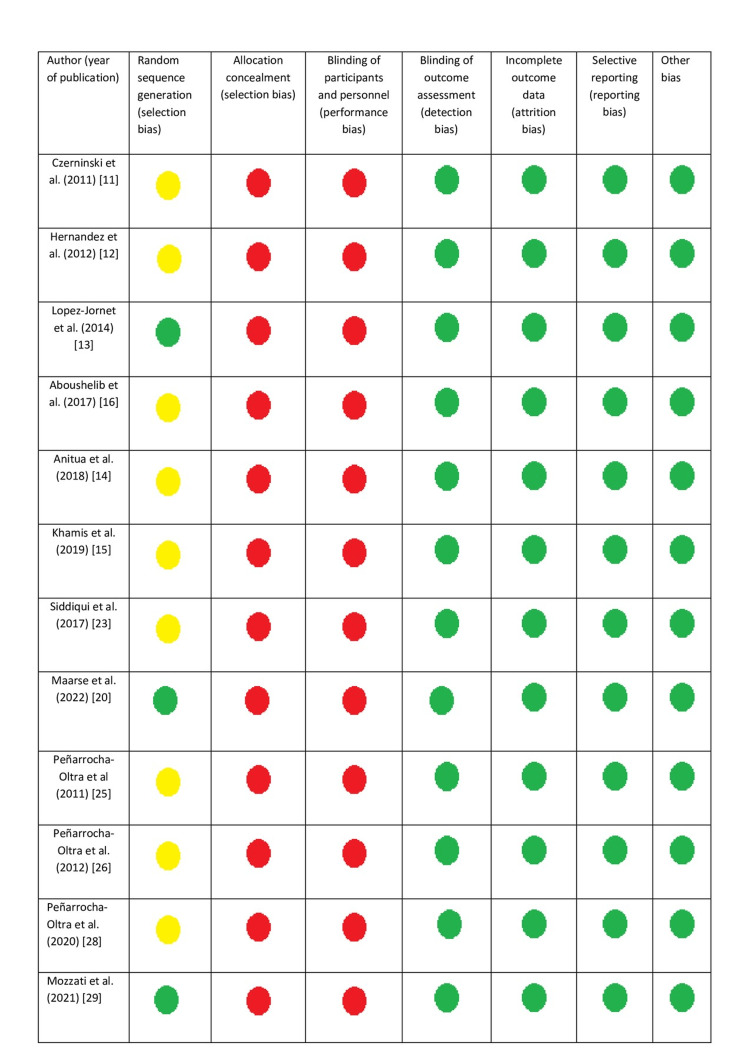
Risk of bias of the included studies Green dot: low risk of bias, red dot: high risk of bias, yellow dot: unclear risk of bias

A modified table of the Cochrane Collaboration for the assessment of the risk of bias was employed to assess the quality of evidence of the selected articles. This table includes seven columns assessing the “risk of bias” (Figure 2). It represents a useful tool to estimate: (1) random sequence generation (selection bias), (2) allocation concealment (selection bias), (3) blinding of participants and personnel (performance bias), (4) blinding of outcome assessment (detection bias), (5) incomplete outcome data (attrition bias), (6) selective reporting (reporting bias), and (7) other bias. Each one of the included articles is judged in these seven categories and labeled as “low risk,” “high risk,” or “unclear risk” of bias. Thus, a summary of the total “risk of bias” can be obtained.

 Treatment outcomes

The data obtained from the included studies are categorized according to autoimmune diseases as follows.

Outcomes Concerning OLP

Six clinical studies on dental implants inserted in patients suffering from OLP were detected and met the inclusion criteria. Czerninski et al. compared clinical manifestations and survival of inserted dental implants between patients suffering from OLP and healthy individuals. The authors evaluated dental implant survival and the potential negative influence on the course of the disease in 29 patients suffering from OLP and found a 100% survival rate for the 54 implants during a 12-24-month follow-up. The authors concluded that the long-term success of dental implants in treated OLP patients did not seem to differ from that of the general population [11]. Moreover, Hernandez et al. surprisingly found a higher rate of survival of endosseous implants (56 implants) placed in patients with OLP (18 patients), compared to the control group (18 patients receiving 62 implants), i.e., 100% and 96.8%, respectively. Peri-implantitis appeared in 10.7% of the implants and 27.7% of the patients with OLP, while peri-implant mucositis was detected in 44.6% of the implants and 66.6% of the same group of patients. The presence of desquamative gingivitis was associated with a higher rate of peri-implant mucositis on the implants of the diseased group [12]. Similar results were also reported by Lopez-Jornet et al. who found no significant differences between OLP patients and the control group with regard to implant survival, peri-implant mucositis, peri-implantitis, and marginal bone loss. The overall success rate in the OLP and control groups was 96.42% and 92%, respectively. The peri-implant mucositis and peri-implantitis prevalence in the OLP group was 17.9% and 25% and in the control group was 18% and 16%. By contrast, they found peri-implantitis to be more frequent in the mandible and in dental implants placed posteriorly compared to those placed anteriorly either in the maxilla or the mandible [13]. Furthermore, a retrospective study by Anitua et al. reported on the use of 66 short implants in 23 patients as an alternative to bone augmentation (≤8.5 mm long). All operations were done outside the flare-up periods of OLP, and a prophylactic regimen of oral corticosteroids was given to avoid flare-ups after the procedure. The investigators did not include a control group for comparison. However, they found a 98.5% survival rate with only one implant out of 66 lost, which occurred in a patient who had erosive OLP with desquamative gingivitis [14]. Khamis et al. evaluated the prognosis of implants placed in 42 OLP patients controlled by low-dose systemic corticosteroids in comparison to noncontrolled patients and a control healthy group during a four-year follow-up. The investigators reported that there was no statistically significant difference in marginal bone loss between healthy and controlled OLP patients. However, noncontrolled patients exhibited a significant increase in marginal bone loss, while recurrence of OLP (erosive and non-erosive types) was confirmed by histopathological evaluation. There were significant interactions between the state of the disease, evaluation time, and the amount of bone resorption observed between different groups [15]. In addition, Aboushelib and Elsafi inserted 55 implants in 23 patients with OLP, with 42 of them failing in a very short period of time - seven to 11 weeks. A new set of 42 implants was then placed and put on a maintenance program for three years under low-dose corticosteroid medication and soft tissue laser irradiation of the patients. The authors reported the positive effect of low doses of corticosteroids in combination with soft tissue laser irradiation [16].

Three case series reporting on patients suffering from OLP were included in the present systematic review. The study of Esposito et al. reveals a 100% survival rate for two patients with erosive OLP who were successfully treated with implant-retained mandibular overdentures (two implants placed per patient) after a follow-up period of 18 months [17]. Similar results are reported in the study of Oczakir et al., which included a subgroup of one patient with OLP where four implants were placed, and a follow-up period of six years, with a survival rate of 100% [18]. Furthermore, Reichart et al. evaluated the performance of 10 implants placed in three patients who developed OLP (asymptomatic and atrophic or mixed atrophic and reticular type). Bone resorption around the implants in all three patients was on average 3-4 mm. The authors recommended a strict follow-up in order to maintain peri implant health [19].

Outcomes Concerning SS

Oczakir et al. included a group of two patients with SS in their study where 12 implants were placed. No complications were observed after two years of function [18]. In the study of Maarse et al., 37 implants were placed in 17 patients with an implant survival rate of 100%, while 26 implants were placed in 17 healthy individuals with a survival rate of 96.2% and a follow-up period of eighteen months. No significant differences in mean marginal bone loss were found between both groups after 18 months of function. Moreover, no significant differences were found for probing depth (SS group: M = 2.29 mm; non-SS group: M = 1.70 mm; p = 0.06) and gingival index (SS group: M = 0.04; non-SS group: M = 0.00; p = 0.42) between the groups, although probing depths were numerically higher in the autoimmune disease group [20]. Korfage et al. compared clinical outcomes in 50 SS patients (primary and secondary forms) with those of healthy patients. In the latter retrospective study, the authors found that the overall survival rate was 97% in the group with SS receiving 140 implants, compared to the healthy population, which showed a survival rate of 100%, in a follow-up period of 46 months. It was observed that the only difference between these two groups was peri-implant mucositis. Specifically, 94% of the SS patient group was diagnosed with peri-implant mucositis versus 62% of the healthy control group. In addition, similar rates of peri-implantitis were diagnosed in both groups. Regarding patients with primary and secondary SS, no difference was found in the prevalence of peri-implant mucositis and peri-implantitis [21]. Moreover, Albrecht et al., in a study based on oral health questionnaires, compared patient-reported answers related to dental implant treatment (outcome, satisfaction, and complications) in 32 SS patients and in healthy control patients. They found a non-significant difference between the two groups - 95.2% survival rate in SS patients and 100% in control patients after a mean period of 4.9 years. The authors recommended implant treatment for patients suffering from SS as a viable option, despite dentists and rheumatologists being reported to advise such patients not to have endosseous implants [22]. Finally, Siddiqui et al. reported a rather low survival rate (87%) of dental implants (23 implants) placed in 11 patients with SS (Siddiqui) in a follow-up period of 40 months. Interestingly, three implants failed in two patients in the second and fifth months after placement. One implant failed due to a lack of osseointegration and the remaining two was removed due to mobility, erythema of the surrounding implant area, vertical bone loss, and horizontal ridge deficiency after implant placement [23].

Outcomes Concerning EB

In the study of Penarrocha-Diago et al., 15 implants were placed and restored successfully in four patients with oral epidermolysis bullosa (OEB). The survival rate was 100% after a mean of two and a half years. The authors observed that implant mucous soft tissues remained in good condition in all cases; no peri-implant bullae were observed. Finally, no significant peri-implant bone loss was recorded [24]. In a retrospective study by David Peñarrocha-Oltra et al., 32 anterior implants were placed in six patients with recessive dystrophic OEB and used to support eight full-arch fixed prostheses; 20 implants were placed in the maxilla and 12 in the mandible. The survival rate was 100% after an average follow-up of 22.9 (range 12-48) months. Intraoperative blister complications were recorded in all cases, although the mucosa remained in good condition around all of the implants, with no peri-implant bullae being observed [25]. Furthermore, in a retrospective study of David Peñarrocha-Oltra/Aloy-Prosper et al., 23 implants were placed in four patients suffering from OEB [25]. Of these 23 implants, 18 implants showed peri-implant defects, 14 were treated with autologous bone, and four received synthetic tricalcium betaphosphate. In 16 implants, resorbable collagen membranes (Lyostypt) were used to protect the particulate bone grafts. The implant survival rate was 100% after a minimum follow-up of 12 months (range 12 to 48). Blister complications were recorded in all cases, especially in relation to implants positioned in the mandible, although no complications related to the bone grafts were observed [26]. Moreover, in a case series of Agustín-Panadero R et al., a total of 31 implants were placed (17 in the maxilla and 14 in the mandible) in four patients (three women and one man) diagnosed with recessive dystrophic OEB who were aged between 20 and 52 years [27]. The implant survival rate after four years was 100% [27]. Finally, in a retrospective study by David Peñarrocha-Oltra/Agustin Panadero 2020 et al., 13 patients were inserted with 80 implants [28]. Two implants failed (2.5%) - one failed during the osseointegration period and the other was removed due to mobility detected 12 months after prosthetic loading. The clinical survival rate of the implants was 97.5%. The authors observed a peri-implant bone loss after 7.7 years of follow-up of 1.65 ± 0.54 mm [28].

Outcomes Concerning LE

The literature search revealed only one publication reporting on patients with LE with or without oral manifestations receiving dental implants (retrospective study) [29]. Mozzati et al. showed that the rehabilitation with calcium ions-modified surface implants (12 implants) associated with plasma rich in growth factors proved to be a safe and effective treatment in five patients with systematic LE. Implant survival at five-year follow-up was 100%. There was one dehiscence reported while mean bone loss was 0.49 mm [29].

Discussion

The present systematic review and analysis of studies aimed to evaluate the impact of autoimmune diseases on the overall biological behavior of dental implants, so that professionals can make improved decisions and refine treatment plans to optimize clinical outcomes. Autoimmune diseases have been assumed to be a risk factor with regard to osseointegration of dental implants. Clinicians are sometimes reluctant when placing implants in such patients, since the stress associated with implant-placement procedures may exacerbate disease manifestations [30]. However, an overall survival rate of 96.2% for implants inserted in systemically compromised patients was calculated in the present review, which is comparable to implant survival rates in healthy individuals. At the same time, patient satisfaction scores on implant treatment were promising across the included studies that evaluated this factor.

Two recent systematic reviews on implants placed in systemically compromised patients have been detected in the literature. Both of the studies suggest that dental implants should be given as a treatment option in patients with autoimmune diseases. Strict follow-up and systemic control of the disease seem to be necessary [31,32]. However, case reports, which are scientifically low evidence, have also been included in the two above-mentioned systematic reviews. No risk of bias assessment and no meta-analysis has been conducted by Esimekara et al., while success rates of implants are being directly compared to survival rates in some cases [31]. Strietzel et al. assessed the risk of bias in included studies through a funnel plot analysis [32].

An interesting finding of the present systematic review was the overall large number of implants identified, which were placed in patients with autoimmune diseases (898 in total). The latter reveals the endeavor of clinicians to treat such cases with implant-supported prostheses, as well as the preference of patients for dental implants as a treatment option.

Peri-implant mucositis and peri-implantitis are common complications reported for systemically compromised patients receiving dental implants. Unfortunately, a further synthesis of data in order to evaluate marginal bone loss rates among the included studies was not possible. Different reference points in measurements among authors did not allow a direct comparison of peri-implantitis and peri-implant mucositis rates, nor a further meta-analysis of this part of the results. In the present systematic review, only articles reporting on dental implants after the year 2000 were included. Previous forms and surface coatings of endosseous implants are not available in the dental market anymore, and therefore it was chosen to only include studies investigating modern types of implants. In addition, the type of prosthesis also plays a crucial role in the long-term performance of dental implants. Fixed prostheses show different mechanical behavior when compared to removable dentures, while the type of abutment material and the type of connection between implant and superstructure may further impact the survival rate of implants. The articles included in the present systematic review were very heterogeneous with regard to the implant prosthesis, and thus no conclusion can be drawn concerning these parameters [33]. Moreover, sex has been shown to impact the course of autoimmune diseases [34], with the prevalence of them being generally higher in women than in men [35]. Unfortunately, the information of gender was not always given in the included studies with regard to implants that were lost among patients. Thus, the possible different outcomes with respect to gender could not be evaluated in the present systematic review.

The overall performance of implants placed in systemically compromised patients according to the findings of the present systematic review is being analyzed for each one of the reported autoimmune diseases below.

Analysis of OLP

Nine clinical studies on OLP were included with a total number of 389 inserted implants, which is a representative sample. In those studies, the success of implant rehabilitation among treated OLP patients was similar to that of healthy individuals, and thus implant treatment seems to be a viable option for such patients. Czerniski et al. concluded that the disease does not influence implant placement, and vice versa - disease manifestations are not being influenced by dental implants. Patients were initially treated with potent steroids and with nonsteroids and antifungal medication during follow-up periods in this study [11]. Hernandez et al. also came to a similar conclusion, with OLP not being associated with a higher prevalence of implant failure or postsurgical complications. Postoperative pain levels were not statistically different in both control and diseased groups. Interestingly, desquamative gingivitis in patients with OLP played a negative role and was statistically significantly associated with peri-implant mucositis [12]. Moreover, Lopez-Jornet et al. concluded that dental implants do not influence the distribution of OLP in the oral cavity [13]. Anitua et al. investigated short implants (<8.5 mm), which are expected to show lower survival rates even in healthy individuals, and documented stable long-term outcomes of those implants in patients with OLP [14]. According to these authors, the clinical type of OLP manifestation seemed to play a role in the long-term behavior of implants with the peri-implant bone being less stable in the erosive type of OLP compared to the reticular type. Interestingly, in the study of Khamis et al., dental implants inserted in patients receiving a low dose of corticosteroids performed better than those placed in patients with uncontrolled-active OLP, underlining thus the importance of proper medication for patients with autoimmune diseases [15]. The latter was also highlighted by Aboushelib et al. in the study of Aboushelib et al., a high failure rate was reported (42 implants failing shortly after surgical insertion), which was attributed by the authors to the active state of OLP. It has not been reported by the authors which clinical type of OLP (erosive, reticular, etc.) the patients presented or if they presented repeated episodes. After treating patients with low-dose corticosteroids and soft tissue laser irradiation, the survival rate of newly inserted implants dramatically improved. The authors emphasized that careful patient selection criteria are crucial when it comes to autoimmune diseases [16]. Finally, in the three case series included in the present systematic review, implants placed in patients with OLP also performed clinically sufficiently [9,17,18].

Analysis of SS

Dental implants placed in patients with SS show a clinical performance comparable to implants inserted in patients without the disease in the short term (95.8% for 316 implants). Patients suffering from SS should be given the option of implant treatment according to the findings of the present systematic review. However, patients with SS showed a susceptibility to soft tissue infection compared with healthy controls. Surprisingly, Maarse et al. reported a higher survival rate for implants placed in patients with SS than in healthy individuals after 18 months of function. The latter underscores the role of motivation in dental hygiene and compliance of the individual itself. Age, educational level, or bad habits, such as smoking, are factors that may influence peri-implant health [20]. Korfage et al. evaluated retrospectively all patients with SS registered in a universal setting and included 50 individuals with 140 implants in their study, which is a large representative sample. Peri-implant mucositis was seen in 94% of those patients compared to 62% in the healthy group, while peri-implantitis was reported for 14% of the patients (11% of the implants) and 12% for the healthy control, indicating an acceptable performance of implants, but still accompanied by mucosal complications [21]. Furthermore, Albrecht et al. included only women in their study and were thus the only authors taking the effect of gender into account in respect of autoimmune diseases. In this study, some of the implants had to be replaced, and the overall survival rate was 95.2% [22]. Finally, Siddiqui et al. reported the lowest survival rate for implants (87%) in patients with SS among the included studies. This investigation though, was based on patient-reported data, and its preliminary goal was to evaluate electronic dental records and not implant treatment itself [23].

Analysis of EB

The findings of the present systematic review suggest that dental implants may be successfully placed in patients with EB with an overall survival rate of 99.5% for the five included studies. Penarrocha Oltra/Diago 2011 et al. inserted a large number of implants (32) for full arch rehabilitation of patients. Full-arch restorations are more demanding, while the distal expansion of the prosthesis performed by the clinicians in this study could overload mucosa. However, no complications or peri-implant bullae were observed [25]. Penarrocha Oltra/Aloy-Prosper et al even placed implants in combination with particulated bone grafts. The authors also reported a survival rate of 100% after a follow-up period of one year [26]. Moreover, Augustin Panadero et al. reported a high patient satisfaction score for the implant treatment and highlighted the benefits of intraoral scanning for patients with mucosal difficulties instead of conventional impression-making [27]. In another study, the importance of improving self-esteem and quality of life for patients with EB through implant treatment was also underlined [28]. However, one point to be taken into consideration is that all five studies on EB included in the present review came from the same investigation group of the University of Valencia, which decreases scientific diversity and increases bias [28].

Analysis of LE

During the search, most case reports on patients with LE treated with implants have been detected and not included in the present review. Only one study was included reporting on implants placed in five patients with LE with or without oral manifestations. There were no failures in the 12 implants placed in those patients. The implants had a calcium ion-modified surface to promote osseointegration. The study’s results are promising, but still the sample is very small to draw any conclusions with regard to LE [29].

Limitations

Studies with different follow-up periods were included in the present review, which is a limitation. However, long-term data with regard to patients with autoimmune diseases receiving implants are very scarce in the literature. Detecting such information with a follow-up period of five or 10 years was unachievable. The overall survival rate of the included studies could be calculated for a follow-up period of one year, which is short term.

Unfortunately, no RCTs could be detected in the literature and the clinical studies included in the present review were prospective, retrospective, and case series. Furthermore, well-designed clinical studies are essential to draw safe conclusions for patients with autoimmune diseases.

## Conclusions

The survival rates of implants placed in patients with autoimmune diseases seem to be satisfactory on the short term - one-year follow-up. However, the disease should be systemically controlled through the appropriate medication. Possible exacerbation of disease manifestations reported in some studies should be taken into consideration. Patients with autoimmune diseases should be offered the option of implant treatment in order to benefit from all the advantages that come with implant-supported prostheses. However, more studies investigating the long-term behavior of implants in such cases should be conducted.
